# Human Leukocyte Antigen (HLA) Alleles Prevent Metabolically-Induced Inflammation and Cerebrocortical Thinning in Gulf War Illness

**DOI:** 10.29245/2572.942x/2020/3.1273

**Published:** 2020-08-20

**Authors:** Peka Christova, Lisa M. James, Adam F. Carpenter, Scott M. Lewis, Brian E. Engdahl, Apostolos P. Georgopoulos

**Affiliations:** 1 Brain Sciences Center, Department of Veterans Affairs Health Care System, Minneapolis, MN, 55417, USA; 2 Department of Neuroscience, University of Minnesota Medical School, Minneapolis, MN 55455, USA; 3 Department of Psychiatry, University of Minnesota Medical School, Minneapolis, MN 55455, US; 4 Department of Neurology, University of Minnesota Medical School, Minneapolis, MN 55455, USA; 5 Department of Psychology, University of Minnesota Medical School, Minneapolis, MN 55455, USA

**Keywords:** Gulf War Illness, Inflammation, Metabolic Human Leukocyte Antigen, Brain Atrophy Persistent Antigens

## Abstract

Independent lines of research have demonstrated that GWI is associated with elevated inflammatory markers, metabolic disruptions, and alterations in brain morphometry. Possessing specific Class II human leukocyte antigen (HLA) alleles, on the other hand, has been shown to protect against GWI and to be inversely associated with symptom severity in a dose-dependent manner. The aim of the present study was to evaluate the association between C-reactive protein (CRP), a marker of inflammation, body mass index (BMI), and brain morphometry in GWI veterans with and without a protective HLA allele. Sixty-three veterans with GWI provided blood samples for evaluation of CRP and HLA, height and weight for calculating BMI, and underwent a 3T magnetic resonance imaging scan from which the volume, surface area, and cortical thickness of 68 cortical regions of interest (ROI) were determined. Results demonstrated that the CRP was highly significantly associated with BMI and cortical thinning in veterans lacking protective HLA alleles but not in those possessing a protective HLA allele. Given the role of HLA in antibody production against foreign antigens, the findings suggest that persistent foreign antigens stemming from lack of immunogenetic protection against them contribute to inflammation, metabolic disruption, and cortical thinning in GWI. The findings are discussed in terms of GW-related exposures that are known to result in inflammation.

## Introduction

Nearly thirty years after the 1990–1991 Persian Gulf War, Gulf War era veterans have continued to suffer from chronic, diffuse symptoms commonly referred to as Gulf War Illness (GWI)^1, 2^. Although respiratory, gastrointestinal, and dermatological systems are involved in the GWI symptom constellation, most of the symptoms such as fatigue, pain, and neurological, cognitive and mood disruptions implicate primarily the central nervous system and the brain, in particular. Consequently, considerable research has investigated the nature and causes of brain abnormalities associated with GWI.

## GWI and brain atrophy

Numerous functional and structural brain abnormalities associated with GWI have been identified^3, 4^. Among the most consistent findings regarding brain abnormalities has been evidence of brain atrophy in veterans with GWI^5–12^. We recently reported substantial (~10%) atrophy in veterans with GWI relative to healthy veterans^5^, in areas proximal to the most permeable regions of the blood brain barrier (BBB)^13^. Disruption of the BBB has been shown to be associated with atrophy in an animal model of GWI in which rats were exposed to a combination of stress restraint and chemicals (nerve agent prophylactics and insecticides) that Gulf War veterans may have encountered in theater^14^. Brain morphology is likely to be compromised by a breakdown in BBB integrity resulting from chronic inflammation^15–17^, which is common in GWI.

## GWI: Lack of immunogenetic protection

Mounting evidence from human^18–21^, in vitro^22–26^, and in silico^27^ studies implicates persistent antigens stemming from lack of immunogenetic protection in GWI and GW-associated inflammation. Specifically, six Class II human leukocyte antigens (HLA), which play an essential role in host protection via antibody production, have been shown to distinguish healthy GW veterans from those with GWI, and the number of these protective HLA alleles corresponds with GWI symptom severity in a dose dependent manner^18^. Furthermore, these protective HLA alleles have been shown to influence brain function in Gulf War veterans^19^ and protect against brain atrophy^21^ characteristic of veterans with GWI^5^. Given the instrumental role of Class II HLA in antibody production against foreign antigens via CD4+ T cells, these studies point to lack of immunogenetic protection against foreign antigens leading to antigen persistence and, consequently, inflammation in veterans with GWI^18, 21^. We have speculated that persistent foreign antigens from vaccines administered to Gulf War veterans lacking immunogenetic protection against them may underlie GWI. To that end, in vitro studies have demonstrated harmful effects of serum from GWI veterans on neural cultures that is ameliorated with serum from healthy Gulf War veterans^22^, pooled antibodies^23^, and antibodies against Gulf War vaccine antigens^24–26^.

## GWI and inflammation

Several studies have documented evidence of inflammation in veterans with GWI^28–31^. C-reactive protein (CRP), a non-specific biomarker of inflammation, has been shown to be elevated in GWI^28–31^, and has been implicated in GWI symptoms involving the brain, in particular^28^. Although historically viewed as a marker of inflammation that arises in response to inflammatory cytokines such as interleukin 6, evidence also suggests that CRP may also play a causal role in inflammation^32^. Thus, CRP may both signal and potentiate inflammation, thereby increasing BBB permeability. Indeed, systemic inflammation has been shown to predict BBB dysfunction^33^. Furthermore, inflammation has been intimately connected to metabolic disorders^34^ which are common among Gulf War veterans^35^.

## Systemic inflammation and neurodegeneration

Considerable evidence suggests a robust association between CRP and chronic inflammatory and neurodegenerative conditions including cardiovascular disease, type 2 diabetes mellitus, age-related macular degeneration, hemorrhagic stroke, Alzheimer’s disease, and Parkinson’s disease^36^. In addition, elevated CRP levels have been associated with brain atrophy even in healthy people^37–41^, highlighting the contribution of inflammatory markers on brain morphology independent of conditions that are typically associated with neurodegeneration. Although the mechanisms remain to be fully elucidated, there is evidence that CRP stimulates production of pro-apoptotic cytokines including interleukin-1β (IL-1β), tumor necrosis factor-α (TNFα), and reactive oxygen species^32^. Elevated peripheral inflammation has been associated with declines in cognitive function, an effect presumed to be mediated by the effect of inflammation on brain morphology^39^. BMI is also inherently related to inflammation because adipose tissues release inflammatory cytokines, which promote further metabolic disruptions in a vicious cycle^34^.

## The current study

Separate lines of research have demonstrated that GWI is associated with elevated CRP, metabolic conditions, and with brain atrophy, and that protection from GWI is conferred by presence of specific Class II HLA alleles. The aim of the present study was to evaluate the association between CRP, body mass index (BMI), and brain morphometry in veterans with GWI with regard to HLA. We have proposed that exposure to foreign antigens coupled with a genetic vulnerability that precludes their elimination results in persistent antigens and, ultimately, inflammation, cell damage, and atrophy^18 ,21, 42^. Recent advances in morphometric analyses permit distillation of cortical volume into its component parts, surface area and cortical thickness, allowing for finer grain examination of the influence of inflammation on these two variables independently.

## Materials and Methods

### Participants

Sixty-three veterans with GWI (59 men and 4 women; age 55.2 ± 1.1 y, mean± SEM) gave informed consent to imaging acquisitions in accordance with the Declaration of Helsinki. GWI status was determined using a self-report symptom checklist that permits classification as GWI case or control according to the Center for Disease Control^1^ and the Kansas criteria^2^, the case definitions recommended by the Institute of Medicine^43^. The Center for Disease Control definition requires one or more symptoms in at least two domains that include fatigue, pain, or mood and cognition. The more restrictive Kansas criteria requires that veterans report moderate to severe symptoms in at least 3 of 6 domains: fatigue, pain, neurological/cognitive/mood, skin, gastrointestinal, and respiratory. All GWI veterans in the present study met both case definitions. Consistent with the Kansas criteria case definition, none of the participants had co-occurring medical or psychiatric conditions that could account for GWI symptoms or impair reporting^2^, including veterans with schizophrenia, bipolar disorder, drug abuse or alcoholism, neurological conditions and inflammatory rheumatological conditions. Individuals with traumatic brain injury were also excluded from the study. The study was approved by the University of Minnesota and Minneapolis VA Health Care System Institutional Review Boards.

### Body Mass Index (BMI)

BMI was computed using the height and weight of the participant (BMI = kg/m^2^).

### CRP

Non-fasting peripheral venous blood samples were collected for evaluation of high sensitivity C reactive protein and analyzed using standard procedures by the Minneapolis VAHCS Clinical Laboratory.

## HLA Genotyping

DNA isolation was carried out from 3 ml of whole blood drawn in EDTA tubes, using a commercially available kit (ArchivePure cat. 2300730) from 5Prime (distributed by Fisher Scientific or VWR) with an expected yield of 50–150μg of DNA. The purified DNA samples were sent to Histogenetics (http://www.histogenetics.com/) for high-resolution HLA Sequence-based Typing (SBT; details are given in https://bioinformatics.bethematchclinical.org/HLA-Resources/HLA-Typing/High-Resolution-Typing-Procedures/ and https://bioinformatics.bethematchclinical.org/WorkArea/DownloadAsset.aspx?id=6482). Their sequencing DNA templates are produced by locus- and group-specific amplifications that include exon 2 and 3 for class I (A, B, C) and exon 2 for class II (DRB1, DRB3/4/5, DQB1, and DPB1) and reported as Antigen Recognition Site (ARS) alleles as per ASHI recommendation^44^.

## Magnetic Resonance Imaging (MRI) acquisition

All data were acquired using a Philips 3T MR scanner (Achieva, Philips Healthcare, Best, The Netherlands). In the initial phase of the study, data were acquired from 18 participants using a phased array SENSitivity Encoding (SENSE) 8-channel head coil for reception. For each participant a high resolution T1-weighted Turbo Field Echo (T1w TFE SENSE) was obtained (168 sagittal slices, TR = 8.1932 ms, TE =3.7520 ms, Acquisition matrix 240 × 240, Flip angel 8 deg., voxel size 0.9375 × 0.9375 × 1 mm). A T2-weighted image (T2w VISTA HR SENSE) was also obtained (180 slices, TR = 2500 ms, TE =363.072 ms, Acquisition matrix 252 × 252, voxel size =0.7813 ×0.7813 × 1 mm). Subsequently, upgrades were applied to the system and data were acquired from the remainder 45 participants using a phased array SENSitivity Encoding (SENSE) 15-channel head coil for reception. For each participant a high resolution T1-weighted Turbo Field Echo (T1w TFE SENSE) was obtained (168 sagittal slices, TR =8.0928 ms, TE = 3.698 ms, Acquisition matrix 240 × 240, Flip angel 8 deg., voxel size 0.7500 × 0.7500 × 1 mm). The T2-weighted (T2w VISTA HR SENSE) was also obtained (168 slices, TR = 2500 ms, TE = 370.346 ms, Acquisition matrix 240 × 240, voxel size = 0.7500 ×0.7500 × 1 mm).

## MRI image processing

Data were processed by a 704-core High Performance Computing system (CentOS 6.5 Linux, Rocks 6.1.1), Matlab R2016 (64 bit), and the Human Connectome Project (HCP; humanconnectome.org) pipeline, including the FreeSurfer (FS; http://surfer.nmr.mgh.harvard.edu) HCP version. Full details were presented previously^5^. Briefly, we used a modified version of FS, implemented in the structural HCP pipeline, which utilizes both T1-w and T2-w images to eliminate uncertainty and improve surface reconstruction^45^.

FS, a widely used tool, provides reliable brain measurements validated by manual^46, 47^ and histological analysis^48^. FS’s surface-based stream reconstructs the cortical surface by using models of the boundary between the white and gray matter and between the gray matter and cerebrospinal fluid (this is the pial surface)^49–51^. This algorithm uses intensity and continuity information to generate a continuous cortical ribbon, and creates a mesh of hundreds of thousands of triangles that recover the geometry and the topology of the pial surface and gray/white boundary. Cortical thickness is calculated as the closest distance from the gray/white boundary and pial surface at each vertex on the tessellated surface in both hemispheres^52^. The FS procedure allows submillimeter resolution that is higher than the voxel resolution of the original data. The cortical ribbon is then parcellated into regions of interests (ROIs) based on gyral and sulcal structures of the Desikan-Killiany atlas^53^. Each resulting image was visually inspected for inaccuracy. Mean thickness, cortical volume, and surface area were extracted for each brain ROI from each individual participant’s brain image.

## Brain areas

The volumes, surface area and thickness of 68 cerebrocortical Regions of Interest (ROIs; 34 left and 34 right) were analyzed. Their names are given in the Appendix.

## Data analysis

For each brain, 68 ROIs × 3 measures (surface, thickness, volume) = 204 values were available, for a total of 63 brains × 204 values/brain = 12852 data values; namely, 4284 values per measure above. For specific groups, N = 68 ROIs × 41 HLA-p0 brains = 2788 values, and N = 68 ROIs × 22 HLA-p1 brains = 1496 values. In addition, for each participant, the following data were available: gender, age, HLA group (presence or absence of a GWI protective allele), BMI, and CRP. CRP values were skewed to the right and were (natural) log-transformed to normalize their distribution^28^. Standard statistical methods were used to analyze the data, including t-test, Fisher’s exact test for 2×2 tables, and partial correlation analyses. All statistical analyses were done using the IBM-SPSS statistical package (version 23).

## Results

### General

Of the 63 participants, 41 (HLA-p0 group) lacked all of the 6 GWI protective alleles (DRB1*01:01, DRB1*08:11, DRB1*13:02, DQB1*02:02, DPB1*01:01, DPB*06:01)^18^, whereas 22 participants (HLA-p1 group) carried one such allele (12 carried DRB1*01:01, 4 DRB1*13:02, 2 DQB1*02:02, 4 DPB1*01:01).

## MRI Acquisition

As mentioned in the Methods above, there were two kinds of MRI acquisitions, one using an 8-channel system and the other a 15-channel system. Participants were distributed similarly In the HLA-p0 group, 13 and 28 participants were imaged with the 8- and 15-channel equipment, respectively, whereas in the HLA-p1 group those counts were 5 and 17, respectively; there was no significant association between the HLA groups and the MRI method of acquisition (P = 0.564, Fisher’s exact test for the 2×2 table).

## Comparisons of non-neural measures between the 2 HLA groups

### Gender.

The HLA-p0 group comprised 37 men and 4 women, whereas the HLA-p1 group comprised 22 men; the two groups did not differ significantly with respect to the gender composition (P = 0.288, Fisher’s exact test for the 2×2 table).

### Age.

The mean ± SEM age for the HLA-p0 group was 54.9 ± 1.5 y, and that for the HLA-p1 group was 55.8 ± 1.6. These means did not differ significantly (t-test, t_[61]_ = 0.378, P = 0.707).

### BMI (Figure 1).

The mean ± SEM BMI for the HLA-p0 group was 31.5 ± 0.82, and that for the HLA-p1 group was 31.0 ± 1.02. These means did not differ significantly (t-test, t_[61]_ = 0.357, P = 0.722).

### CRP (Figure 2).

CRP was appreciably lower in the HLA-p1 group but the difference did not reach statistical significance: The mean ± SEM ln(CRP) for the HLA-p0 group was 0.81 ± 0.15, and that for the HLA-p1 group was 0.44 ± 0.20. These means did not differ significantly (t-test, t_[61]_ = 1.46, P = 0.149).

### Association of CRP and BMI (Figure 3).

In the HLA-p0 group, ln(CRP) was positively and significantly correlated with BMI (r = 0.409, P = 0.006, N = 41). In contrast, there was no significant correlation in the HLA-p1 group (P = 0.309).

## Comparisons of association of CRP with neural measures between the 2 HLA groups

The association of CRP with neural measures (cortical volume, surface and thickness) was evaluated by computing the partial correlation coefficient between ln(CRP) and each measure above, controlling for estimated total intracranial volume (eTIV), age and gender, as needed (e.g. gender was entered as covariate only for the HLA-p0 group, since the HLA-p1 group comprised only men). We found the following.

### Association of CRP with cortical volume.

No statistically significant partial correlations (r_p_) were found between ln(CRP) and cortical volume in either HLA group (HLA-p0, controlling for age, gender, and eTIV: r_p_ = −0.009, P = 0.645, DF = 2783; HLA-p1, controlling for age and eTIV: r_p_ = −0.009, P = 0.722, DF = 1492).

### Association of CRP with cortical surface.

No statistically significant partial correlations were found between ln(CRP) and cortical surface in either HLA group (HLA-p0, controlling for age, gender, and eTIV: r_p_ = −0.002, P = 0.903, DF = 2783; HLA-p1, controlling for age and eTIV: r_p_ = −0.018, P = 0.405, DF = 1492).

### Association of CRP with cortical thickness (CT) (Figure 4).

A highly statistically significant partial correlation was found between ln(CRP) and cortical thickness in the HLA-p0 group (r_p_ = −0.063, P = 0.00092, DF = 2783, controlling for age, gender, and eTIV). In contrast, no significant partial correlation was found for the HLA-p1 group (r_p_ = 0.045, P = 0.081, DF = 1492, controlling for age and eTIV; notice the positive correlation). A more detailed examination of the ln(CRP) vs. thickness relation in the HLA-p0 group revealed that a better fit was a power fit, i.e. a linear relation in a log-log scale. This makes sense, since it implies that even very high levels of CRP will never result in zero thickness. The results for the log-log relation were: r_p_ = −0.07, P = 0.000208, DF = 2783, controlling for age, gender, and eTIV). The association of cortical thickness (lnCT) and CRP (lnCRP) remained significant after accounting for BMI (r_p_ = −0.069, P = 0.000284, DF = 2782, controlling for BMI, age, gender, and eTIV). BMI was not significantly associated with cortical thickness in either group (HLA-p0 group: r = −0.023, P = 0.215, N = 2788; HLA-p1 group: r = −0.037, P = 0.157, N = 1496).

### Comparison of cortical thickness between the two HLA groups.

Cortical thickness, adjusted for age, gender, eTIV, and ln(CRP) was overall higher in the HLA-p1 than the HLA-p0 group but this difference did not reach statistical significance; the mean ± SEM of ln(CT) for the HLA-p0 group was 0.911 ± 0.003, N = 41, and for the HLA-p1 group 0.916 ± 0.005, N = 22; P = 0.346).

## Discussion

Here we investigated the effect of GWI-protective HLA alleles on the association between inflammation, BMI, and brain morphometry in veterans with GWI. The results demonstrated a highly significant association between CRP with BMI and cortical thickness, but not surface area or cortical volume, in GWI veterans lacking one of the protective Class II HLA alleles. There were no such associations in veterans possessing one of the protective alleles. These findings suggest that persistent antigens stemming from lack of HLA protection influence metabolic and inflammatory systems, contributing to cortical thinning. These results add to the literature highlighting the role of inflammation in GWI, narrow the focus of GWI risk factors to those that are known to contribute to inflammation, and point towards potential treatment avenues aimed at reducing inflammation. We discuss below GW-related exposures, highlighting the potential role of persistent antigens on GWI inflammation.

## Differential Associations of CRP on Brain Morphometry

Several studies have documented morphometric alterations associated with GWI including reduced brain volume^4, 5^. Here we focused on the cerebral cortex, analyzing its volume, surface area, and thickness. The current study documents the association between CRP and reduced cortical thickness in GWI veterans lacking protective HLA alleles, but no association with surface area or cortical volume. This dissociation of effects is consistent with findings that changes in cortical gray matter volume are almost exclusively driven by changes in surface area^54, 55^, and that surface area and volume are underlain by distinct genetic influences^56^ and reflect different aspects of brain architecture and distinct cellular mechanisms^55^. In addition, cortical thickness and surface area are negatively correlated^57^. Cortical thickness is a more sensitive measures of gray matter alteration than volume^58^, and many neurological and psychiatric conditions are associated with variations in cortical thinning^59–64^, leading some to suggest cortical thickness is a candidate biomarker for differential diagnosis and disease prognosis^65^. The current study provides evidence of cortical thinning in veterans with GWI who lack immunogenetic protection; longitudinal studies are warranted to examine the association of cortical thickness with GWI disease prognosis.

## Metabolic and Immune System Interactions and Cortical Thinning

Considerable research highlights a robust association between the immune and metabolic systems such that inflammation is unequivocally causally associated with metabolic system disruptions which promote further inflammation^34^. Indeed, the association between inflammation and obesity has been referred to as a “chicken or the egg” question as activation of the immune system can promote weight gain and obesity, and obesity can perpetuate inflammation^34^. Furthermore, CRP and BMI have each been linked to reduced cortical thickness^66, 67^, as has expression of genes specific to astrocytes and microglia, the brain’s resident immune cells^68^. Here, CRP, a marker of inflammation, was highly significantly associated with BMI and cortical thinning, but only in veterans lacking a protective HLA allele; BMI was not associated with cortical thinning in either group. This suggests that persistent antigens resulting from lack of immunogenetic protection against them may contribute to both inflammation and obesity, the latter of which may promote additional inflammation. In contrast, there were no significant associations between CRP, BMI, and cortical thickness in GWI veterans possessing 1 protective HLA allele. Taken together, these findings raise the possibility of at least two inflammatory drivers in GWI – one stemming from persistent antigens and a separate mechanism that includes metabolically-induced inflammation. It also raises the possibility that the varied symptoms associated with GWI may be partially attributable to differences in HLA composition. For example, cognitive symptoms may be more prominent in those lacking HLA protection due to reduced cortical thickness stemming from persistent antigens. Next, we discuss environmental exposures that may contribute to antigen persistence in GWI, highlighting the role of vaccines.

## In-theater exposure and GWI

GWI is typically attributed to deployment-related exposures either alone or in combination with psychological stress or other exposures. Commonly investigated in-theater exposures includes chemical nerve agents (e.g., sarin) and widespread use of pyridostigmine bromide (PB) to protect against possible nerve agent attacks, frequent use of pesticides, and airborne contaminants from oil well fires. While implicating in-theater exposures has provided convenient explanations for the excess symptomatology seen in GWI veterans, relative to non-deployed GW veterans or those deployed to other regions, the lack of documentation on actual in-theater exposures limits causal connections regarding exposure to any specific agent and GWI. Furthermore, despite decades of study it remains unclear how exposure to such toxicants could contribute to GWI symptoms 30 years later. Take, for instance, sarin. A number of military personnel were exposed to low-level sarin following the destruction of an Iraqi ammunition dump, in Khamisiyah, Iraq. Although sarin is extremely toxic, it is short-lived inside the body where it is quickly metabolized and excreted through urine. Thus, while some deleterious effects may be expected following sarin (and other toxicant exposure), it is unclear how sarin could contribute to progressive effects nearly 30 years later given the rapid metabolism and short half-life. A condition known as organophosphate-induced chronic neurotoxicity (OPICN) has been described in the literature, and is characterized by neuropathological, neurological, neuropsychological, and neurobehavioral alterations as a result of a large single exposure or repeated small sarin exposures^69^. Notably, the characteristics are similar to those seen in GWI and some have considered OPICN as a possible explanation for GWI^70^. OPICN may indeed be related to GWI, particularly the excess of GWI seen in deployed veterans relative to their non-deployed and other-deployed counterparts; however, to date, the mechanisms of OPICN progressive effects remain unclear^71^ and the contribution of sarin and other nerve agents to GWI is largely inconclusive. Others have concluded that pesticides and/or PB are causally associated with GWI^4, 72^. Similar to nerve agent exposure, use of pesticides and PB may play a contributory role in GWI symptomatology; however, neither OPICN, pesticides, nor use of PB can account for GWI cases observed in non-deployed veterans. It is notable that up to 36% of veterans who never deployed (depending on which GWI case definition was used) report GWI-consistent symptom profiles^4, 72^, suggesting that the constellation of symptoms referred to as GWI cannot be solely attributable to in-theater exposure.

## Vaccines and GWI

Both deployed and non-deployed GW veterans received multiple vaccinations in preparation for the war and those vaccines have become another widely investigated risk factor for GWI. Reports indicate that new recruits during the GW era received up to 17 vaccines during the first two weeks of basic training^72^. Several studies have concluded that vaccinations are associated with GWI, and have documented evidence of GWI in non-deployed GW veterans and in other-deployed veterans who received vaccines, albeit at lower rates than deployed GW veterans^2, 73, 74^. Furthermore, a dose-response relationship has been reported between the number of vaccines and severity of health symptoms^31^. In addition to standard vaccines (e.g., yellow fever, cholera, hepatitis B, etc.), the Gulf War was the first time that a large number of military personnel (150,000 deployed and non-deployed veterans) received the anthrax vaccine, the safety of which has been called into question^75^. As Nass^75^ discusses, an unusually high rate of adverse events was reported for anthrax vaccine relative to other vaccines, with vaccine recipients developing at least 2 of the following 3 symptoms: fatigue, muscle or joint pains, and cognitive or emotional impairment, symptoms consistent with the CDC case definition of GWI^1^. Nass^75^ concludes that statements that existing scientific evidence does not support an association between anthrax vaccine and GWI are simply not true. Indeed, given the incidence of GWI in non-deployed veterans who received preparatory vaccinations, it seems quite likely that anthrax and potentially one or more of the 17 antigens administered are implicated in GWI. To that end, recent in vitro studies have documented deleterious effects of GWI serum on cell cultures and reversal of those effects with the concomitant addition of antibodies against antigens contained in the vaccines administered to GW veterans^24–26^.

## Vaccines, Persistent Antigens, and Inflammation

In discussing the role of vaccines in GWI, the Institute of Medicine^76^ noted that adverse effects of vaccinations could be due to the toxicity of the antigen in the vaccine and/or from stimulation of the immune system (the intended goal of immunization) that may result in immune enhancement, hypersensitivity, or an immune-mediated pathological response. They continue to describe acute effects (e.g., rash) as well as sustained effects including tissue damage due to inflammation that tends to be seen in the lung, kidney, joints, and brain in animal studies.

Finally, the IOM report notes that genetic inheritance strongly influences the immune response to immunization and to actual infection, and explains the high variability in response to vaccinations. HLA genes, in particular, are highly related to immune response. Antibody production hinges on a match between epitopes derived from an antigen and an individual’s HLA profile. In the absence of an HLA-antigen match, the antigen persists^21, 42^, contributing to inflammation and ultimately tissue damage. As previously mentioned, there is mounting evidence documenting inflammation in veterans with GWI^28–31^. Furthermore, we identified 6 HLA alleles that were present in healthy GW veterans but were absent or significantly less frequent in those with GWI, indicating that GWI is associated with genetic lack of protection^18^. Thus, we suspect that GWI is a result of persistent antigens that could not be eliminated due to HLA-antigen incongruence, resulting in inflammation and ultimately neurodegeneration. Notably, HLA variability has been specifically implicated in regards to variability in antibody production to anthrax vaccine^77^. Similarly, in silico analyses highlight variability in the binding affinity of GWI vaccine antigens to protective HLA, thereby differentially affecting antibody production^27^.

We have studies underway aimed at identifying persistent antigens in veterans with GWI with the goal of ultimately eliminating them (thereby reducing inflammation and slowing progression of cortical thinning) via personalized immunotherapy. Findings from two recent in vitro studies in our lab demonstrated that serum from veterans with GWI results in detrimental changes to cell morphology in neural cultures; however, the damaging effects were neutralized with the addition of serum from healthy Gulf War veterans^22^ or human immunoglobulin G^23^. Consistent with the Persistent Antigen hypothesis^21, 42^, the neutralizing effects are presumably due to ability of antibodies present in serum from healthy Gulf War veterans and in pooled IgG to eliminate persistent antigens in veterans with GWI. Subsequent in vitro studies have documented evidence of adverse of GWI serum on cell cultures and reversal of those effects with the concomitant addition of antibodies against antigens contained in the vaccines administered to GW veterans^24–26^. Taken together, these findings highlight the fact that harmful persistent antigens circulating in the blood partly contribute to inflammation and ultimately cortical thinning in GWI. We anticipate that elimination of persistent antigens would reduce those effects.

## Limitations

The findings from the present study must be considered in light of limitations. First, a small number of HLA alleles were investigated in the present study; thus, the effects of other HLA alleles on metabolically-induced inflammation and cerebrocortical thinning are unknown. The six Class II HLA alleles investigated here were identified from among 144 Class I and Class II alleles in a prior study of GWI^18^. These six alleles were shown to discriminate veterans from GWI from healthy GW controls, and exhibited a dose-dependent relationship with GWI symptoms such that fewer protective alleles were associated with more GWI symptoms. Other alleles did not confer that protection and are, therefore, considered non-protective. Subsequent studies have demonstrated various protective effects of these alleles as it relates to GWI^19–27^. This study extends those findings to demonstrate additional protection of these six Class II HLA alleles on metabolically-induced inflammation and cerebrocortical thinning. Second, the veterans in the present study had elevated BMI which is known to be associated with inflammation via release of cytokines^34, 78, 79^; however, there were no difference in BMI between veterans with and without protective alleles, and BMI did not account for the observed association between CRP and cerebrocortical thinning in veterans without protective HLA alleles. There is the possibility that this group of GW veterans with elevated BMI are not representative of GWI veterans in general although that is unlikely given that several other studies of Gulf War veterans have similarly found elevated BMI^30, 31, 80, 81^. Finally, the study sample was relatively small. Future studies with larger samples are warranted to corroborate the findings and evaluate generalizability to the broader GWI population.

## Conclusion

In the present study we demonstrated that inflammation is highly associated with BMI and cortical thinning in veterans with GWI who lack protective HLA alleles, but not in those who possess a GWI-protective HLA allele. These findings suggest that inflammation due to the persistence of foreign (potentially vaccine-related) antigens resulting from lack of HLA protection contributes to GWI. Future studies aimed at eliminating persistent antigens may be effective in reducing the varied symptoms associated with GWI, particularly those involving the brain.

## Figures and Tables

**Figure 1.**
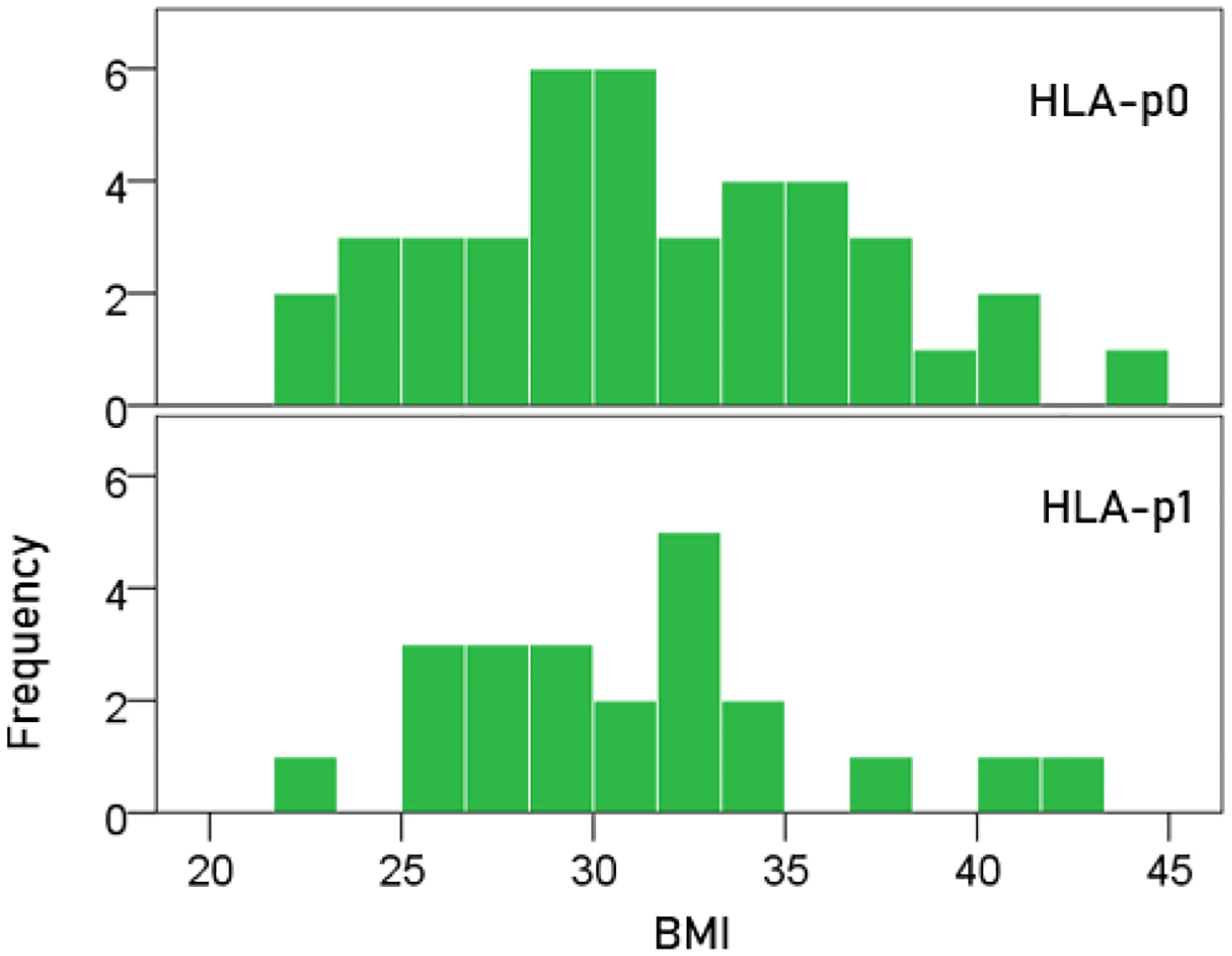
Frequency distribution of BMI for the two HLA groups. (See text for details.)

**Figure 2.**
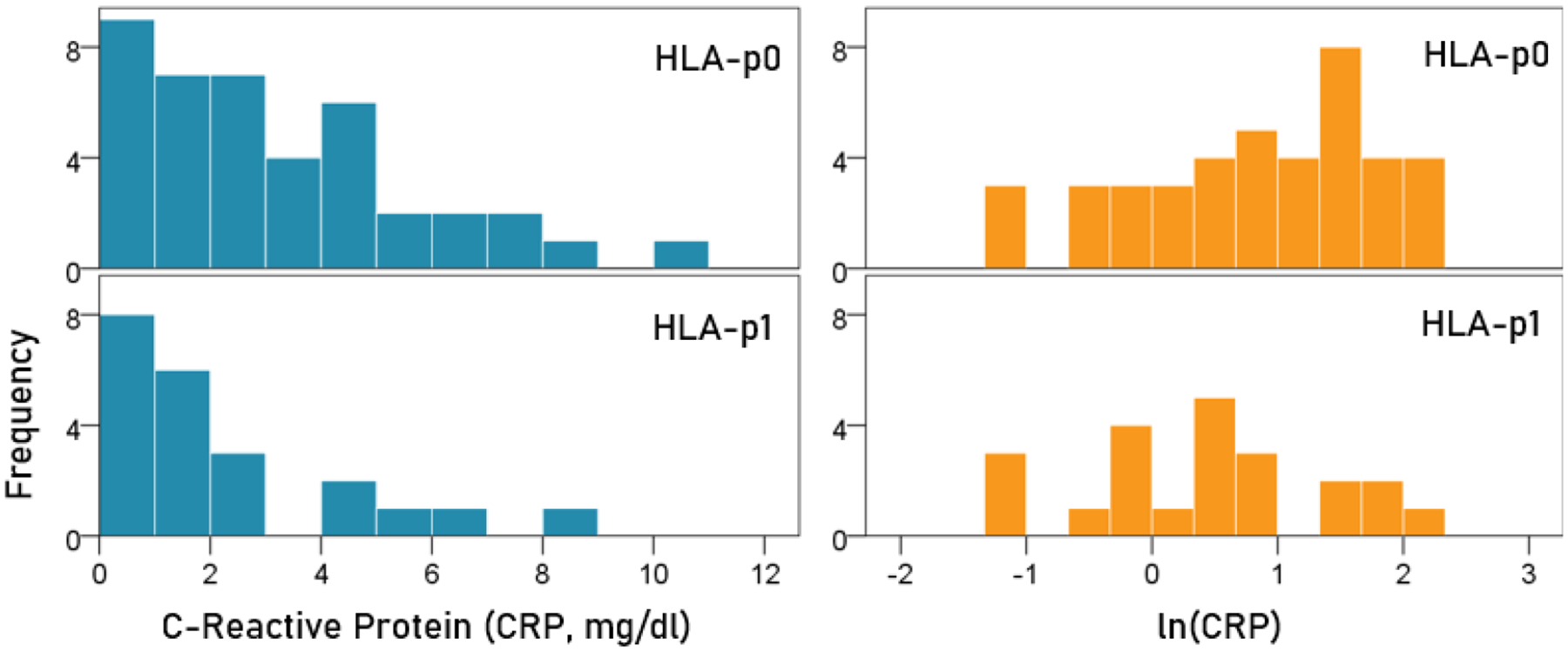
Frequency distribution of CRP and ln(CRP) for the two HLA groups. (See text for details.)

**Figure 3.**
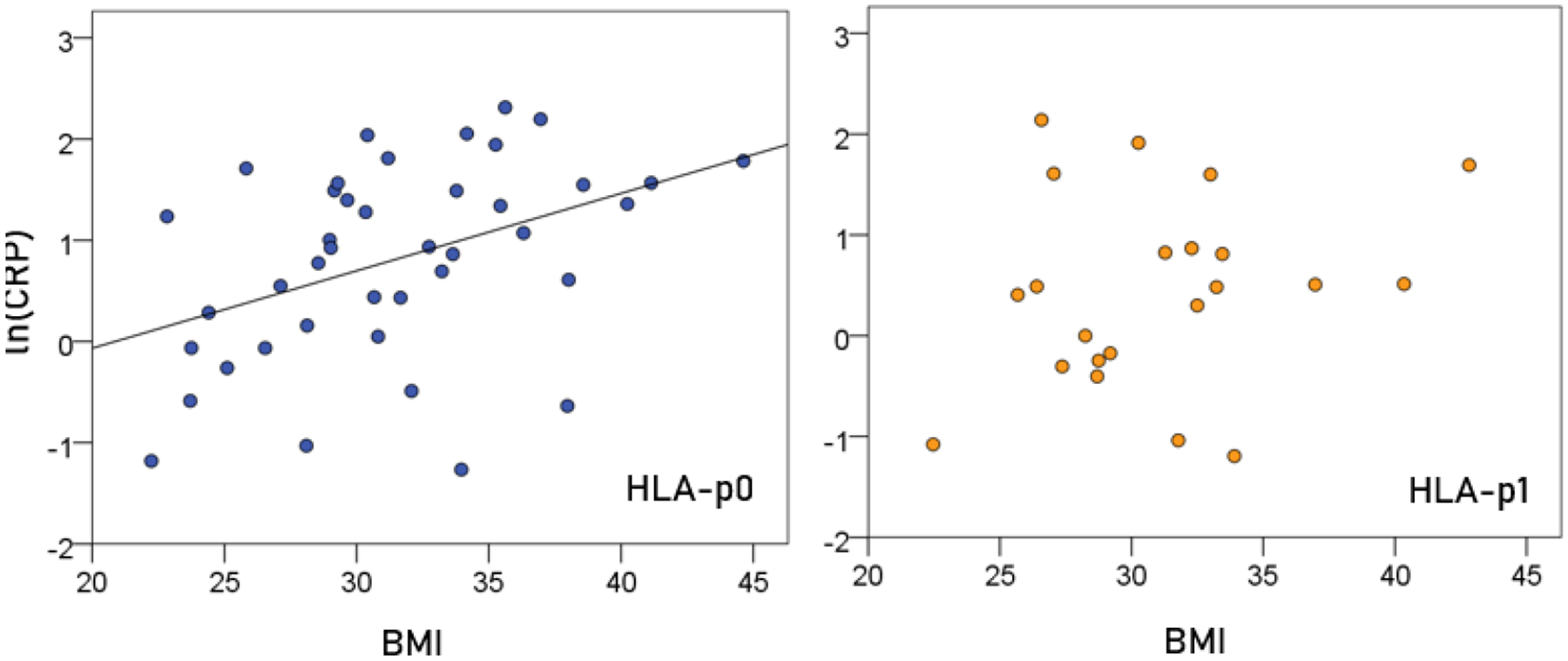
ln(CRP) is plotted against BMI for the two HLA groups. (See text for details.)

**Figure 4.**
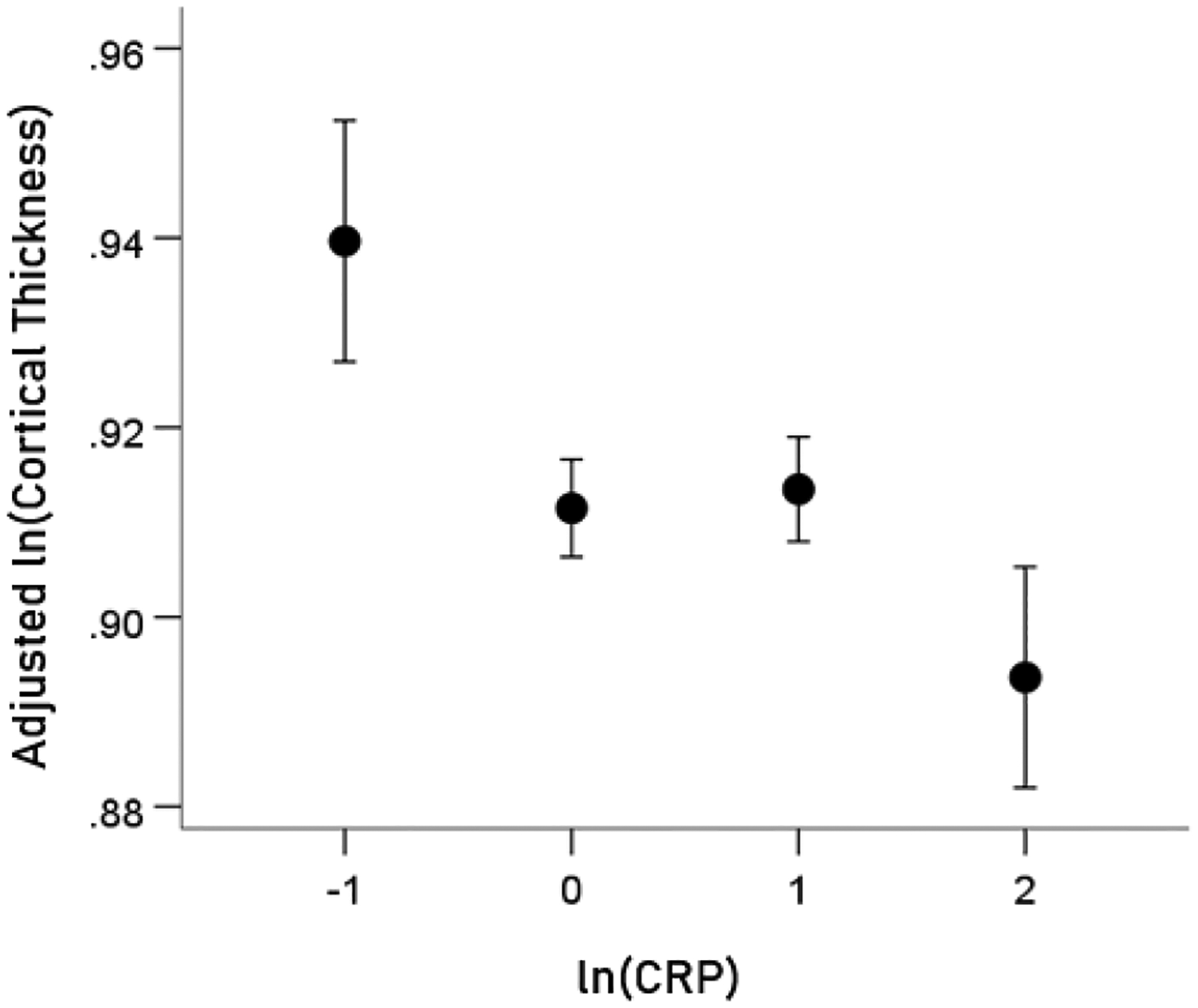
HLA-p0 group: ln(Cortical thickness) adjusted for age, gender and eTiV (mean ± SEM) is plotted against binned ln(CRP) (binwidth = 1 lnCRP unit). (See text for details.)

## Appendix Cortical ROIs used

**Table:**

1	Left banks of the superior temporal sulcus
2	Left caudal anterior cingulate cortex
3	Left caudal middle frontal gyrus
4	Left cuneus cortex
5	Left entorhinal cortex
6	Left fusiform gyrus
7	Left inferior parietal cortex
8	Left inferior temporal gyrus
9	Left isthmus-cingulate cortex
10	Left lateral occipital cortex
11	Left lateral orbitofrontal cortex
12	Left lingual gyrus
13	Left medial orbitofrontal cortex
14	Left middle temporal gyrus
15	Left parahippocampal gyrus
16	Left paracentral lobule
17	Left inferior frontal gyrus pars opercularis
18	Left inferior frontal gyrus pars orbitalis
19	Left inferior frontal gyrus pars triangularis
20	Left pericalcarine cortex
21	Left postcentral gyrus
22	Left posterior cingulate cortex
23	Left precentral gyrus
24	Left precuneus cortex
25	Left rostral anterior cingulate cortex
26	Left rostral middle frontal gyrus
27	Left superior frontal gyrus
28	Left superior parietal cortex
29	Left superior temporal gyrus
30	Left supramarginal gyrus
31	Left frontal pole
32	Left temporal pole
33	Left transverse temporal cortex
34	Left insula
35	Right banks of the superior temporal sulcus
36	Right caudal anterior cingulate cortex
37	Right caudal middle frontal gyrus
38	Right cuneus cortex
39	Right entorhinal cortex
40	Right fusiform gyrus
41	Right inferior parietal cortex
42	Right inferior temporal gyrus
43	Right isthmus-cingulate cortex
44	Right lateral occipital cortex
45	Right lateral orbitofrontal cortex
46	Right lingual gyrus
47	Right medial orbitofrontal cortex
48	Right middle temporal gyrus
49	Right parahippocampal gyrus
50	Right paracentral lobule
51	Right inferior frontal gyrus pars opercularis
52	Right inferior frontal gyrus pars orbitalis
53	Right inferior frontal gyrus pars triangularis
54	Right pericalcarine cortex
55	Right postcentral gyrus
56	Right posterior cingulate cortex
57	Right precentral gyrus
58	Right precuneus cortex
59	Right rostral anterior cingulate cortex
60	Right rostral middle frontal gyrus
61	Right superior frontal gyrus
62	Right superior parietal cortex
63	Right superior temporal gyrus
64	Right supramarginal gyrus
65	Right frontal pole
66	Right temporal pole
67	Right transverse temporal cortex
68	Right insula
